# A grave complication: propylthiouracil-induced antineutrophil cytoplasmic antibody-associated vasculitis

**DOI:** 10.1210/jcemcr/luag009

**Published:** 2026-02-24

**Authors:** Christopher Christopoulos, Tiffany Mach, Vanessa Tardio, George Tsoukas

**Affiliations:** Faculty of Medicine and Health Sciences, Université de Montréal, Montreal, QC, Canada H3T 1J4; Endocrinology and Metabolism Residency Training Program, Department of Medicine, McGill University, Montreal, QC, Canada H3A 0G4; Division of Endocrinology and Metabolism, Department of Medicine, McGill University Health Centre (MUHC), Montreal, QC, Canada H4A 3J1; Division of Endocrinology and Metabolism, Department of Medicine, McGill University Health Centre (MUHC), Montreal, QC, Canada H4A 3J1

**Keywords:** propylthiouracil, hyperthyroidism, Graves disease, vasculitis

## Abstract

Propylthiouracil (PTU) is a commonly used antithyroid agent for the treatment of hyperthyroidism. While generally well tolerated, it has been associated with serious adverse effects, including agranulocytosis, hepatotoxicity, and, more rarely, antineutrophil cytoplasmic antibody (ANCA)-associated vasculitis. We present a case of a 58-year-old woman, known for Graves disease on 5 years of PTU treatment, who developed a hypertensive emergency and progressive renal dysfunction, initially attributed to nonsteroidal anti-inflammatory drug use. Further laboratory evaluation and kidney biopsy confirmed a diagnosis of ANCA-associated vasculitis likely induced by long-term PTU treatment. PTU was discontinued, and a prednisone taper was initiated, resulting in improvement in renal function. Following PTU discontinuation, corticosteroid therapy, and blood pressure management, the patient entered a euthyroid state with improved renal function.

## Introduction

Graves disease, the most common cause of hyperthyroidism, is treated with antithyroid drugs such as methimazole or propylthiouracil (PTU), radioactive iodine, or thyroidectomy. Although PTU is generally well tolerated, it has been associated with rare but serious adverse effects, including agranulocytosis, hepatotoxicity, and vasculitis. This case highlights an atypical presentation of PTU-induced antineutrophil cytoplasmic antibody (ANCA)-associated vasculitis occurring years after drug initiation, which was initially misattributed to nonsteroidal anti-inflammatory drug toxicity, thus delaying diagnosis and treatment.

## Case presentation

A 58-year-old woman with a known diagnosis of Graves disease and rheumatoid arthritis treated with adalimumab, presented to the emergency department with a hypertensive crisis. Prior to her presentation to the hospital, she noted persistently elevated systolic blood pressures >170 mmHg with new onset of a pulsatile headache with a “thudding” sensation in her ears. These symptoms were associated with a 6-month history of fatigue.

The patient was diagnosed with Graves disease in 2015 and was initially treated with methimazole. However, the medication was discontinued in 2020 due to a drug-induced rash. She was subsequently transitioned to PTU 50 mg per os (PO) twice a day. Her longstanding rheumatoid arthritis had been initially treated with etanercept; however, this was discontinued in 2014 due to corneal melting. She was then changed to adalimumab in addition to naproxen 500 mg PO daily as needed for her joint pains. Her family history was notable for rheumatoid arthritis in both parents.

## Diagnostic assessment

Clinically, the patient only experienced a persistent headache. She denied any dyspnea, dizziness, syncope, chest pain, visual changes, nausea, vomiting, or abdominal pain. On presentation, she was afebrile with a blood pressure of 150/73 mmHg, heart rate 81 beats per minute, respiratory rate 18 per minute, and oxygen saturation 96% on room air. Physical exam was unremarkable except for bilateral lower extremity edema. There were no associated neurological deficits or constitutional symptoms.

Complete blood count, electrolytes, and liver enzymes were within normal limits, TSH was elevated at 13 mU/L (SI: 13 mIU/L) (normal reference range 0.38-5.33 mU/L; [SI: 0.38-5.33 mIU/L]), and free T4 was within normal limits at 0.78 ng/dL (SI: 10 pmol/L) (normal reference range 0.54 to 1.24 ng/dL; [SI: 7.0-16.0 pmol/L]). Her creatinine was elevated at 1.50 mg/dL (SI: 133 μmol/L) (normal reference range 0.60-1.20 mg/dL [SI: 53-106 μmol/L]) with an estimated glomerular filtration rate 39 mL/min (normal estimated glomerular filtration rate >60 mL/min) from a normal baseline, indicating acute kidney injury. Abdominal computed tomography scan revealed no acute intra-abdominal pathology. An abdominal ultrasound demonstrated right and left kidneys measuring 12.5 and 12.4 cm, respectively (normal kidney size is 9-13 cm in females, 10-14 cm in males). Benign renal cysts were identified on ultrasound and were not considered clinically significant; therefore, no additional investigations were pursued.

## Treatment

Based on these initial investigations, the patient was started on intravenous hydration. It was suspected that she most likely had nonsteroidal anti-inflammatory drug-induced nephrotoxicity given her acutely worsening renal function with normal complete blood count, electrolytes, and liver function. Imaging revealed no evidence of obstruction or masses. Therefore, naproxen was discontinued, and she was started on amlodipine and bisoprolol for further optimization of her blood pressure. She was subsequently referred to nephrology for outpatient management.

## Outcome and follow-up

Despite these initial interventions, her renal function continued to decline over the following 2 months with her creatinine rising to 3.17 mg/dL (SI: 280 μmol/L). Her ANCA serology came back positive, raising suspicion for a renal vasculitis. A kidney biopsy was subsequently done, which demonstrated segmental sclerosis and fibrocellular crescents, compatible with an ANCA-induced vasculitis.

Given that PTU was the most recently introduced medication, it was identified as the likely trigger. Therefore, PTU was discontinued and a prednisone taper was initiated starting at 75 mg PO daily for 1 week, then 40 mg PO daily for 1 week, then 30 mg PO daily for 2 weeks, then 25 mg PO daily for 2 weeks, then 20 mg PO daily for 2 weeks, then 15 mg PO daily for 2 weeks, then 10 mg PO daily for 2 weeks and finally 2.5 mg PO daily for 1 year.

At 4-month follow-up, the patient was clinically and biochemically euthyroid. Her TSH level was 4.43 mU/L (SI: 4.43 mIU/L), and creatinine improved to 1.62 mg/dL (SI: 143 μmol/L) while on her prednisone taper. Her blood pressure was well controlled after the addition of perindopril. She was found to have cushingoid features, including a round and plethoric face, mild proximal muscle weakness, and ecchymoses, presumably due to prolonged steroid use over the prior year.

## Discussion

Although rare, PTU-induced ANCA-associated vasculitis is a serious and potentially life-threatening complication. The kidneys are most commonly affected; however, the lungs, skin, and joints may also be involved [1]. New-onset fever, hypertension, dyspnea, hematuria, decreased urine output, or rash should raise suspicion for a vasculitis. In patients receiving PTU, prompt recognition and initiation of management are critical as the disease can progress rapidly.

The mechanism of PTU-induced ANCA-associated vasculitis is not fully understood. One proposed pathway involves PTU metabolites being transformed by neutrophil myeloperoxidase, which triggers a lymphocyte-mediated immune response [1]. Another theory involves neutrophil extracellular traps, which expose a DNA-protein structure that traps pathogens that are later digested by the granulocytes. PTU may alter neutrophil extracellular trap conformation, promoting production of antibodies such as ANCA [2]. Only a subset of patients with ANCA-positive antibodies develop vasculitis clinically, likely influenced by an underlying genetic predisposition and environmental factors, such as silica exposure and upper respiratory tract infections [3, 4].

PTU-induced vasculitis typically occurs in women between the ages of 30 and 60 years and often emerges weeks to months after treatment initiation [5]. Patients with additional nonthyroidal autoimmune diseases, such as rheumatoid arthritis, are more susceptible due to their immune hypersensitivity, thus increasing the risk of vascular injury. In our patient, the combination of the patient's sex, age, medical comorbidities, and PTU exposure created a predisposition for developing ANCA-induced nephritic vasculitis.

In most cases, discontinuation of the offending drug is sufficient in the management of vasculitis secondary to PTU. However, given our patient's increased risk for complications, including progressive renal injury due to multiple comorbidities and delayed diagnosis, steroid therapy was added to mitigate systemic inflammation and preserve renal function. The diagnosis was complicated by the late onset of vasculitis, which occurred years after PTU initiation, as well as the initial misdiagnosis of naproxen toxicity. Consequently, the diagnosis and appropriate management were delayed, allowing for significant progression of nephritic damage. Even though kidney function and blood pressure often normalize after discontinuing PTU, our patient required several months of antihypertensive therapy and immunosuppressive treatment. The prolonged course was likely attributable to a substantial delay in reaching the correct diagnosis. This case emphasizes the importance of early recognition of PTU-induced ANCA vasculitis. Timely intervention may have mitigated the extent of renal injury, reduced morbidity, and prevented the need for prolonged corticosteroid therapy and its associated complications.

In conclusion, this case highlights a rare but important adverse effect of PTU, specifically ANCA-induced renal vasculitis. While effective in the management of hyperthyroidism, PTU carries significant risks requiring ongoing surveillance, including hepatic dysfunction, agranulocytosis, and vasculitis, even years into treatment. In patients presenting with inflammatory symptoms on antithyroid therapy, it is important to consider a diagnosis of PTU-induced vasculitis with prompt investigations, notably ANCA testing and renal biopsy, if there is renal dysfunction. Physicians should educate their patients on the risk of these inflammatory symptoms and encourage them to seek emergency care if they develop. Early recognition and discontinuation of PTU, alongside appropriate immunosuppressive therapy, are critical in preventing irreversible organ damage.

## Learning points

- PTU-induced vasculitis can occur insidiously and may present several years after treatment initiation.
- Clinicians should maintain a high index of suspicion for PTU-induced vasculitis in patients presenting with unexplained systemic or inflammatory symptoms, particularly when renal involvement is suspected.
- Early recognition and prompt evaluation of PTU-induced vasculitis with ANCA serologies and kidney biopsy are key for preventing irreversible organ damage, especially progression to chronic kidney disease or end-stage renal disease.

## Contributors

All authors made individual contributions to authorship. C.C. and T.M. were involved in the acquisition of data and drafting the manuscript. V.T. and G.T. were involved in the diagnosis and management of the patient. All authors reviewed and approved the final version of the manuscript.

## Data Availability

Data sharing is not applicable to this article as no datasets were generated or analyzed during the current study.
